# Inequities in Dementia Diagnosis: Evidence From the ELSI‐Brazil Study

**DOI:** 10.1002/gps.70219

**Published:** 2026-04-25

**Authors:** Andrew Christopher Claro Miguel, Lucas Martins‐Teixeira, Carolina Godoy, Gustavo Melo de Andrade Lima, Maria Fernanda Lima‐Costa, Matheus Ghossain Barbosa, Cleusa Pinheiro Ferri

**Affiliations:** ^1^ Department of Psychiatry Escola Paulista de Medicina Universidade Federal de Sao Paulo Sao Paulo Brazil; ^2^ Research Unit Responsabilidade Social ‐ Hospital Alemão Oswaldo Cruz São Paulo Brazil; ^3^ Neurology Division Escola Paulista de Medicina Universidade Federal de Sao Paulo Sao Paulo Brazil; ^4^ Center of Studies in Public Health and Aging Fundação Oswaldo Cruz e Universidade Federal de Minas Gerais Belo Horizonte Brazil

**Keywords:** Brazil, dementia, inequities, LMICs, underdiagnosis

## Abstract

**Objective:**

To estimate the national proportion of undiagnosed dementia cases in Brazil, examine its distribution across Brazilian regions sociodemographic subgroups, and identify factors associated with receiving a diagnosis.

**Methods:**

We conducted a cross‐sectional, population‐based analysis using baseline data (2015–2016) from the Brazilian Longitudinal Study of Aging (ELSI‐Brazil), a nationally representative survey of community‐dwelling adults. Dementia was identified through an established algorithm incorporating cognitive testing and functional impairment, combined with self‐reported medical diagnosis of Alzheimer's disease. Underdiagnosis was defined as meeting dementia criteria without a prior medical diagnosis. Sociodemographic, clinical, cognitive, and functional variables were assessed. Survey‐weighted logistic regression models estimated factors associated with underdiagnosis.

**Results:**

Among 5249 participants aged ≥ 60 years, 392 met criteria for dementia. Overall, 83.1% (95% CI: 76.5–88.1) had no previous diagnosis. Underdiagnosis was more frequent in poorer regions (90.2%) than in richer regions (76.0%), and higher among illiterate individuals (93.9%). In fully adjusted models, older age (OR = 0.91; 95% CI: 0.85–0.97), more years of education (OR = 0.86; 95% CI: 0.76–0.96), a higher number of chronic conditions (OR = 0.72; 95% CI: 0.54–0.97), and better memory performance (OR = 0.68; 95% CI: 0.56–0.84) were associated with a lower likelihood of underdiagnosis, while living alone was associated with a higher likelihood of underdiagnosis (OR = 3.65 [1.05–12.6]).

**Conclusions:**

About four in five older Brazilians meeting dementia criteria had no prior clinical diagnosis, with marked sociodemographic and regional disparities. Both individual factors—such as age, education, and multimorbidity—and structural inequities across regions influenced diagnostic likelihood. Strengthening early‐detection strategies, improved health professional training, and regionally tailored approaches may improve recognition of dementia in Brazil's public health system.

## Key Points

- About four in five older Brazilians identified as having dementia had no prior medical diagnosis.
- Underdiagnosis was markedly higher in socioeconomically disadvantaged regions compared with wealthier regions.
- Healthcare utilization alone was not associated with diagnosis, suggesting that routine service contact does not translate into dementia‐specific screening or referral.
- Higher education, greater multimorbidity, and better cognitive performance were associated with an increased likelihood of reporting a previous diagnosis.

## 1 Introduction

Dementia is one of the fastest‐growing global public health challenges, affecting an estimated 57.4 million people in 2019 and projected to reach 152.8 million by 2050 [1]. This rapid increase is particularly pronounced in low‐ and middle‐income countries (LMICs), where nearly two‐thirds of all cases are expected to occur [2, 3]. In Brazil, approximately 2.5 million people were estimated to be living with dementia in 2025, a number expected to triple by 2050 as demographic transitions accelerate [4]. Despite this growing burden, gaps in diagnosis and care remain substantial.

Timely and accurate diagnosis—occurring when symptoms first prompt clinical attention and functional impairment remains limited—is essential for clinical management, patient and caregiver education, care planning, and access to treatments and psychosocial support [5, 6]. However, underdiagnosis of dementia is widespread globally. Studies from high‐income countries show that a substantial proportion of individuals meeting diagnostic criteria never receive a formal diagnosis. A comparative analysis conducted 10 years apart across 19 European countries found persistently high underdiagnosis rates, including 86.6% in Croatia and 85.2% in Portugal [7]. In LMICs, underdiagnosis may exceed 90% [8], driven by structural, socioeconomic, and cultural barriers—including limited awareness, stigma, restricted access to specialized health services, insufficient training of healthcare professionals, and fragmented care pathways [9].

In Brazil, additional challenges further hinder timely diagnosis. The belief that cognitive decline is an expected consequence of aging remains prevalent, even among healthcare providers, contributing to delayed or missed diagnoses [10]. Empirical evidence on dementia underdiagnosis in the country is scarce and geographically restricted: two studies from São Paulo, the wealthiest Brazilian state, reported underdiagnosis rates of 77% estimated from local dispensing data of cholinesterase inhibitors [11] and 71% from outpatient internal medicine clinics [12]. More recently, the 2024 National Dementia Report from the Brazilian Ministry of Health, using national medication dispensing data, suggested persistently high underdiagnosis rates nationwide which was potentially higher in socioeconomically disadvantaged macroregions [13]. This raises the possibility that underdiagnosis reflects underlying regional inequities in socioeconomic conditions and access to healthcare. Yet, no population‐based studies to date have formally examined these disparities or the factors associated with them.

Understanding the magnitude and distribution of dementia underdiagnosis is essential for guiding public health action. Brazil is characterized by marked regional inequities in income, educational opportunities, and healthcare access [14, 15]—all factors that may influence diagnostic likelihood. Identifying which sociodemographic, clinical, cognitive, and functional characteristics are associated with underdiagnosis can support the development of targeted strategies and inform national health systems, training programs, resource allocation, and early detection initiatives.

Using data from the Brazilian Longitudinal Study of Aging (ELSI‐Brazil), a nationally representative study of community‐dwelling older adults, this study aimed to estimate the prevalence of dementia underdiagnosis in Brazil, examine its distribution across sociodemographic subgroups, and identify factors associated with receiving or not receiving a clinical diagnosis of Alzheimer's disease among individuals who met criteria for probable dementia.

## 2 Methods

### 2.1 Study Design and Participants

This was a cross‐sectional, population‐based analysis using baseline data from the first wave of the Brazilian Longitudinal Study of Aging (ELSI‐Brazil). ELSI‐Brazil is a nationally representative household survey of community‐dwelling adults aged ≥ 50 years and serves as the Brazilian partner study of the international Health and Retirement Study (HRS) [16]. Baseline data were collected in 2015–2016 across 70 municipalities covering all five Brazilian geopolitical regions. The sampling strategy combined stratification and multistage clustering to ensure representativeness of the Brazilian aging population. All residents aged ≥ 50 years in sampled households were eligible for individual interviews and physical assessments, which were conducted through standardized home‐based procedures administered by trained interviewers [17]. Of the 9412 completed interviews, 5249 participants aged ≥ 60 years had complete data for dementia classification and were included in the present analyses. Further details can be seen elsewhere [17].

### 2.2 Definition of Underdiagnosed Dementia

Dementia classification was derived using a previously published algorithm developed specifically for ELSI‐Brazil's participants aged 60 and above [18], combined with self‐reported medical diagnosis of Alzheimer's disease. The algorithm relied on the cognitive assessments collected in ELSI‐Brazil, which evaluated five cognitive domains: temporal orientation (day, month, year, weekday), semantic verbal fluency (1‐minute animal naming), episodic memory (immediate and delayed recall from a 10‐word list), prospective memory (delayed instruction recall), and semantic memory (four everyday/political knowledge items). Performance in each domain was standardized into z‐scores, and their mean constituted the global cognitive score.

To derive regression‐based norms, a normative subsample was constructed following the original protocol, excluding individuals with characteristics that may impair cognitive performance: hearing or visual impairments affecting test execution; previous diagnosis or symptoms of depression (CES‐D ≥ 4) [19]; history of Alzheimer's disease, Parkinson's disease, or stroke; heavy alcohol consumption based on National Institute on Alcohol Abuse and Alcoholism criteria [20]; self‐ or informant‐reported memory complaints; functional impairment in ≥ 1 of four gender‐independent instrumental activities of daily living (IADLs: managing money, using transportation, using the telephone, taking medications); and missing data for exclusion criteria.

Within this normative subsample, regression‐based norms were generated to predict global cognitive scores based on age, sex, and education. Standardized residuals (observed minus predicted, divided by the residual SD) were computed for all participants. Cognitive impairment was defined as a residual global cognitive *z*‐score ≤ −1.5.

Participants were classified as having dementia when cognitive impairment (*z* ≤ −1.5) was accompanied by functional impairment, defined as difficulty in ≥ 1 of the four IADLs used in the algorithm, or by an IQCODE score ≥ 3.4. Additionally, participants who self‐reported a medical diagnosis of Alzheimer's disease (“Has a doctor ever told you that you have Alzheimer's disease?”) were classified as having dementia regardless of algorithm results.

Underdiagnosed dementia was defined as fulfilling the dementia case definition described above while not reporting a previous medical diagnosis of Alzheimer's disease.

### 2.3 Other Variables

Sociodemographic characteristics included age (years), sex (male/female), years of education, living arrangement (living alone vs. living with others), and geographic region grouped according to Brazil's macro‐regional socioeconomic development: the Southeast and South—responsible for approximately 53% and 17% of the national gross domestic product (GDP), respectively—were grouped as richer regions, whereas the North, Northeast, and Central‐West—accounting for roughly 5%, 14%, and 10% of GDP—were grouped as poorer regions [21].

Clinical variables included the total number of chronic conditions, derived from self‐reported physician diagnoses of hypertension, diabetes, high cholesterol, myocardial infarction, angina, heart failure, stroke, asthma, chronic obstructive pulmonary disease, arthritis, osteoporosis, chronic back pain, cancer, and chronic kidney disease (range 0–14). Access to health services was defined as self‐reported contact with any healthcare service (family doctor or specialist consultation, emergency department visit, or hospital admission) within the 2 weeks preceding the interview.

Cognitive performance was assessed using semantic verbal fluency (total number of animals named correctly in 1 minute), and the memory total score—composed of the sum of correct immediate and delayed recalls from the 10‐word list learning task. Functional status was assessed using basic activities of daily living (BADLs) and IADLs, through standardized self‐reported interviews, with assistance from a proxy informant when the participant was unable to respond independently. BADLs included seven items: crossing a room, dressing, bathing, eating, rising from bed, toileting, and continence. IADLs included 10 items: personal hygiene, preparing a hot meal, managing money, using transportation, shopping, using a telephone, taking medication, performing light household tasks, and performing heavy household tasks. Each item was scored using a 4‐point Likert scale (1 = no difficulty; 2 = slight difficulty; 3 = great difficulty; 4 = unable to perform, requires assistance). BADL and IADL scores were calculated as the sum of their respective items, with higher scores reflecting greater functional impairment.

### 2.4 Statistical Analysis

We estimated the proportion of dementia cases that were undiagnosed overall and across sociodemographic strata, and compared general characteristics of diagnosed and underdiagnosed individuals using chi‐square tests or *t*‐tests, as appropriate. Confidence intervals for proportions were calculated using a logit‐transformed Wald method. Multivariable logistic regression models were then applied to identify sociodemographic, clinical, cognitive, and functional factors associated with underdiagnosis. Model 1 included only sociodemographic characteristics (age, sex, education, living alone and living in poorer/richer country regions), model 2 included all variables in model 1 plus the number of chronic conditions and health services use, and model 3 included all variables in model 2 plus cognitive function (verbal fluency and memory total score) and functionality (ABVDs and AIVDs). Results are presented as odds ratios (ORs) with 95% confidence intervals (95% CI). All analyses accounted for the complex sampling design of ELSI‐Brazil using survey‐weighted procedures (svyset) in Stata/SE 17.0 (StataCorp, College Station, TX), ensuring nationally representative estimates.

## 3 Results

**FIGURE 1:**
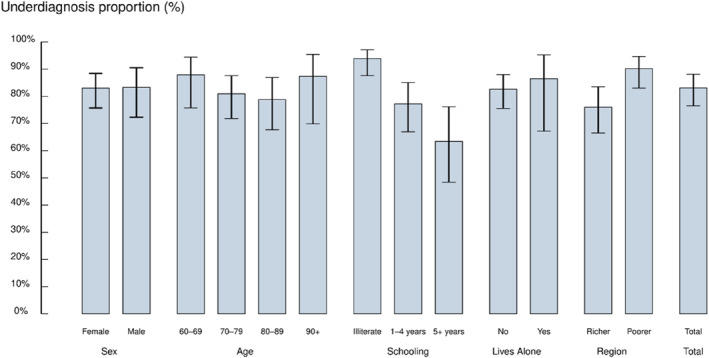
Proportion of underdiagnosed dementia cases across sociodemographic subgroups.

Among the 5249 participants aged ≥ 60 years, 392 participants were identified as having dementia by the algorithm and/or had self‐reported previous diagnosis. Their mean age was 77.2 years (SD = 9.5), two‐thirds were women (67.9%), and nearly half were illiterate (49.6%). Compared to those with a diagnosis, people with dementia without a diagnosis were slightly younger (76.9 vs. 79.2), had a higher proportion of illiteracy (54.6% vs. 22.6%) and were more likely to live in the poorer regions of the country (63.3% vs. 40.3%) (Table 1). Overall, 83.1% (95% CI: 76.5–88.1) of dementia cases were undiagnosed. The proportion of underdiagnosis was highest among those aged 60–69 years (87.9%) and lowest in the 80–89‐year group (78.8%), increasing again among the oldest old (87.4%). Underdiagnosis was particularly elevated among illiterate individuals (93.9%) and was substantially more frequent in poorer regions (90.2%) compared to richer regions (76.0%) (Figure 1).

**TABLE 1:** Sociodemographic characteristics of participants with dementia, stratified by diagnosis status.

	Total dementia (*n* = 392)	Without diagnosis (*n* = 327)	With diagnosis (*n* = 62)	*t*‐test/chi‐square (*p* value)
Sex, *n* (%)				
Female	266 (67.9)	224 (68.5)	41 (66.1)	0.713
Male	126 (32.1)	103 (31.5)	21 (33.9)	
Age, mean (SD)	77.2 (9.5)	76.9 (9.6)	79.2 (8.6)	0.039
Age group, *n* (%)				
60–69	95 (24.2)	84 (25.7)	10 (16.1)	0.178
70–79	136 (34.7)	113 (34.6)	22 (34.5)	
80–89	119 (30.4)	93 (28.4)	25 (40.3)	
90+	42 (10.7)	37 (11.3)	5 (8.1)	
Education (years) mean (SD)	2.2 (3.1)	4.1 (3.6)	1.9 (2.9)	< 0.001
Education, *n* (%)				
Illiterate	193 (49.6)	177 (54.6)	14 (22.6)	< 0.001
1–4 years	143 (36.8)	113 (34.9)	29 (46.8)	
5+ years	53 (13.6)	34 (10.5)	19 (30.6)	
Living alone, *n* (%)				
No	338 (86.2)	278 (85.0)	58 (93.5)	0.073
Yes	54 (13.8)	49 (15.0)	4 (6.4)	
Regions, *n* (%)				
Richer	160 (40.8)	120 (36.7)	37 (59.7)	0.001
Poorer	232 (59.2)	207 (63.3)	25 (40.3)	

In multivariate analyses, sex was not associated with underdiagnosis across all models. Higher education was inversely associated with underdiagnosis in all models. Residing in poorer regions was associated with higher odds of underdiagnosis in Models 1 (OR = 2.63; 95% CI: 1.20–5.76) and 2 (OR = 2.57; 95% CI: 1.12–5.88), but this association was attenuated after adjusting for cognitive and functional variables. In the fully adjusted model, older age (OR = 0.91; 95% CI: 0.85–0.97), more years of education (OR = 0.86; 95% CI: 0.76–0.96), a higher number of chronic conditions (OR = 0.72; 95% CI: 0.54–0.97), and better memory performance (OR = 0.68; 95% CI: 0.56–0.84) were significantly associated with lower odds of underdiagnosis, while living alone (OR = 3.65 [1.05–12.6]) was associated with a higher likelihood of underdiagnosis (Table 2).

**TABLE 2:** Sociodemographic and clinical factors associated with dementia underdiagnosis.

	Model 1	Model 2	Model 3
	OR (95% CI)	OR (95% CI)	OR (95% CI)
Sex (male)	1.40 (0.65–3.02)	1.60 (0.63–4.07)	1.50 (0.39–5.65)
Age (years)	0.97 (0.93–0.99)	0.96 (0.93–1.00)	0.91 (0.85–0.97)
Education (years)	0.83 (0.74–0.93)	0.83 (0.75–0.92)	0.86 (0.76–0.96)
Living alone	2.36 (0.66–8.36)	2.12 (0.63–7.16)	3.65 (1.05–12.6)
Poorer regions	2.63 (1.20–5.76)	2.57 (1.12–5.88)	3.54 (0.97–12.88)
Number of chronic conditions		0.86 (0.70–1.07)	0.72 (0.54–0.97)
Health services access		2.85 (0.70–11.68)	1.29 (0.43–3.89)
Fluency total score			0.95 (0.85–1.07)
Memory total score			0.68 (0.56–0.84)
BADL total score			1.06 (0.78–1.42)
IADL total score			1.08 (0.94–1.25)

## 4 Discussion

In this population‐based study—the first to provide nationally representative estimates of dementia underdiagnosis among Brazilians aged 60 and above, we found that 83% of individuals meeting criteria for dementia had no prior medical diagnosis, underscoring a substantial detection gap at the population level. Underdiagnosis varied markedly across the country: it reached 90% in the poorer regions and was comparatively lower (76%) in the richer ones. These patterns reflect longstanding structural inequities that shape access to diagnostic opportunities and the responsiveness of the health system across Brazil.

The magnitude of underdiagnosis observed in our study is broadly consistent with international evidence showing that missed or delayed dementia diagnosis is a major global challenge. Nonetheless, the proportion identified in Brazil appears higher than what is typically reported in high‐income countries, where estimates average around 54% in Europe and 63% in North America, although some Asian settings report rates approaching 90% [8]. Notably, no comparable estimate was available for Latin America. The overall underdiagnosis rate we observed (83%) also exceeds earlier Brazilian estimates for the state of Sao Paulo (71% [12] and 77% [11]) and is consistent with projections from the 2024 National Dementia Report [13], which estimated national underdiagnosis between 80% and 89% based on indirect indicators derived from patterns of acetylcholinesterase inhibitor dispensing within the public health system. Although these figures were produced using different methodological approaches, both converge with our findings from a nationwide sample, suggesting that a substantial proportion of dementia cases in Brazil remain unidentified by health services.

For the first time, the association between socioeconomic disparities and the proportion of undiagnosed dementia have been documented using country‐level data. Living in poorer macroregions was associated with substantially higher underdiagnosis. This effect was attenuated in the fully adjusted model, suggesting that part of the regional gap is mediated by differences in cognitive performance and functional status at the time of assessment. These findings may also reflect deeper systemic inequities—such as differences in the quality of education, continuity of care, and the organization and capacity of local health services. However, the absence of an association with healthcare utilization suggests that contact with services alone is insufficient to ensure diagnosis.

Diagnostic opportunity depends not only on contact with the health system, but also whether cognitive changes are actively recognized, evaluated, and communicated during clinical encounters. Although healthcare utilization itself was not associated with diagnosis, multimorbidity was; individuals with a higher number of chronic conditions had lower odds of remaining undiagnosed. This may reflect that multimorbidity leads to more frequent and longitudinal interactions with clinicians, increasing opportunities for subtle cognitive changes to be observed [22]. Moreover, when cognitive or behavioral changes do not confirm to the prototypical Alzheimer's disease presentation, clinicians may be less likely to suspect dementia, highlighting the need for broader training in primary care on the diversity of dementia subtypes [22]. Furthermore, the broader context of daily care may also influence diagnostic likelihood. People with dementia who have multiple chronic conditions often require regular attention and structured care routines. In this regard, a population‐based study from the United States found that 60% of nursing‐home residents had received a prior diagnosis, compared with only 37% of those living at home [23]. Similarly, a systematic review reported that approximately 51% of nursing‐home residents with dementia were undiagnosed, compared with 64% among community‐dwelling older adults [8]. These patterns support the hypothesis that social oversight and day‐to‐day support—beyond formal healthcare encounters—may increase the recognition of cognitive decline. This aligns with our finding that living alone was associated with higher underdiagnosis.

This third‐party perspective is particularly important when cognitive symptoms are not yet so prominent, or less noticeable in an overall picture. Nonetheless, stigma and limited awareness surrounding dementia—among families, communities, and even healthcare professionals—may further discourage timely evaluation and contribute to missed recognition [24, 25]. In this study, underdiagnosis was more frequent both among the younger older adults (60–69 years) and the oldest old. Lower recognition in the younger segment may relate to reduced clinical suspicion and limited use of cognitive screening [26] in this age range, where memory complaints are less expected and other conditions often take diagnostic priority. Conversely, the rise in underdiagnosis among the oldest old may reflect a combination of diagnostic deprioritization, misattribution of symptoms to “normal aging,” and the influence of factors such as frailty, accumulated comorbidities, and functional decline, which can complicate clinical evaluation and delay dementia identification [27].

However, while symptom visibility and clinical suspicion shape whether dementia is recognized by others, the likelihood of reporting a prior diagnosis also depends on the individual's own cognitive and educational resources. In our study, better memory performance was associated with higher odds of having received a diagnosis. This pattern may partly reflect recall bias: individuals with better episodic memory are more capable of accurately remembering and reporting a previous medical diagnosis. It may also reflect conceptual understanding, as stronger semantic abilities can facilitate comprehension of medical terminology—such as what it means to be diagnosed with “Alzheimer's disease.” Moreover, those with better cognitive performance are likely to be at earlier stages of decline, when awareness of cognitive changes is greater and anosognosia is less frequent, increasing the chances that they or their families seek evaluation. Educational attainment may reinforce these processes. Higher schooling—consistently associated with lower underdiagnosis across all models—likely enhances both cognitive reserve (i.e., the brain's capacity to tolerate neuropathology while maintaining cognitive function) and health literacy, improving individuals' ability to recognize cognitive changes as pathological and to navigate the healthcare system to obtain timely assessment [28].

This study has strengths, including the use of a nationally representative sample, and the integration of sociodemographic, clinical, cognitive, and functional information to examine correlates of underdiagnosis. The ability to assess regional disparities offers an additional perspective that is seldom available in studies from LMICs. Several limitations should also be acknowledged. Dementia classification combined an algorithmic approach with self‐reported medical diagnosis, which may lead to misclassification. Specifically, participants were asked about a diagnosis of Alzheimer's disease, while the algorithm identified all‐cause dementia; this definitional mismatch could overestimate underdiagnosis. Self‐reported diagnosis is also susceptible to recall and reporting biases, particularly among those with poorer cognitive performance. Additionally, although the ELSI‐Brazil algorithm employed regression‐based norms adjusted for age, sex and education—reducing the risk of misclassifying low‐education individuals as cognitively impaired—residual educational bias cannot be entirely excluded, particularly among illiterate individuals. Furthermore, functional status was assessed through self‐report which may be influenced by social desirability bias, particularly for personal care items, leading to the underestimation of functional impairment. Finally, the cross‐sectional design limits causal interpretation, underscoring the need for longitudinal analyses to better understand diagnostic pathways over time.

## 5 Conclusions

In this nationally representative study, more than four out of five older Brazilians who met criteria for dementia had no prior clinical diagnosis, underscoring a substantial gap in detection within the healthcare system. Marked regional and sociodemographic differences indicate that underdiagnosis is not uniformly distributed, but instead reflects structural inequities in access to diagnostic opportunities, health literacy, and service responsiveness across the country. Factors associated with receiving a diagnosis ‐including age, educational attainment, living alone and number of chronic conditions—suggest that both individual characteristics and system‐level dynamics influence diagnostic likelihood.

Together, these findings highlight the need for strengthened early‐detection strategies within primary care, improved public and professional awareness, and regionally tailored policies to reduce diagnostic disparities. Expanding training, screening capacity, and care pathways—particularly in socioeconomically disadvantaged regions—may help reduce disparities in recognition over time. Future research should explore diagnostic trajectories longitudinally, assess how health system organization influences recognition, and evaluate potential interventions to support earlier identification of dementia in diverse real‐world settings.

## Author Contributions

We confirm that all listed authors have contributed to this manuscript, and that every significant contributor has been included. Andrew Christopher Claro Miguel wrote the first draft, contributed to conceptualizing, the data analysis and interpretation, reviewing and submitting processes. Lucas Martins‐Teixeira, Carolina Godoy, Gustavo Melo de Andrade Lima and Matheus Ghossain Barbosa contributed to the writing, reviewing/data interpretation. Maria Fernanda Lima‐Costa led the cohort design and data collection and contributed to writing and reviewing. Cleusa Pinheiro Ferri supervised and contributed to conceptualizing, data analysis, writing, reviewing, and submitting. All authors have read and approved the manuscript.

## Funding

The ELSI‐Brazil was supported by the Brazilian Ministry of Health: DECIT/SCTIE (Grants 404965/2012‐1, TED 28/2017, and TED 4/2022) and COPID/DECIV/SAPS (Grants 20836, 22566, 23700, 25560, 25552, 27510, and TED 32/2022). M.F.L.C. and C.P.F. are recipients of research productivity fellowships from the National Research Council (CNPq).

## Ethics Statement

The study was approved by the Research Ethics Committee of the Fundação Oswaldo Cruz—Gerais (protocol numbers 34649814.3.0000.5091). Participants signed informed consent forms before taking part in the study.

## Conflicts of Interest

The authors declare no conflicts of interest.

## Data Availability

Data from the Brazilian Longitudinal Study of Aging (ELSI‐Brazil) are available upon request at the ELSI‐Brazil project website (https://elsi.cpqrr.fiocruz.br/).
